# Achieving Presence through Evoked Reality

**DOI:** 10.3389/fpsyg.2013.00086

**Published:** 2013-02-26

**Authors:** Jayesh S. Pillai, Colin Schmidt, Simon Richir

**Affiliations:** ^1^Arts et Metiers ParisTechAngers, France; ^2^Le Mans UniversityLe Mans, France

**Keywords:** presence, reality, dream, virtual reality, simulated reality, cognition

## Abstract

The sense of “Presence” (evolving from “telepresence”) has always been associated with virtual reality research and is still an exceptionally mystifying constituent. Now the study of presence clearly spans over various disciplines associated with cognition. This paper attempts to put forth a concept that argues that it’s an experience of an “Evoked Reality (ER)” (illusion of reality) that triggers an “Evoked Presence (EP)” (sense of presence) in our minds. A Three Pole Reality Model is proposed to explain this phenomenon. The poles range from Dream Reality to Simulated Reality with Primary (Physical) Reality at the center. To demonstrate the relationship between ER and EP, a Reality-Presence Map is developed. We believe that this concept of ER and the proposed model may have significant applications in the study of presence, and in exploring the possibilities of not just virtual reality but also what we call “reality.”

## Introduction

Research on presence has brought to our understanding various elements that certainly cause or affect the experience of presence in one way or another. But in order to evoke an illusion of presence, we in effect try to generate an illusion of reality different from our apparent (real world) reality through different mediations like Virtual Reality. The attempt to evoke an illusory reality is what brought researchers to think about presence in the first place. “Reality,” despite its being a major concept, is most often either overlooked or confused with other aspects that affect presence. To study presence we must first understand the reality evoked in one’s mind. It is this illusion of reality that forms a space-time reference in which one would experience presence. It is evident from the research in the field of virtual reality, that if a medium is able to create a convincing illusion of reality, there will certainly be a resultant feeling of presence. Various theories have been proposed, to explore and define the components of this mediated presence. We aim to abridge those theories in an efficient manner. Moreover, studies in the field of cognition and neuroscience confirm that the illusion of reality can as well be non-mediated (without the help of external perceptual inputs), that is purely evoked by our mind with an inception of corresponding presence. One of the most common but intriguing example of a non-mediated illusion of reality would be – a dream. This self evoking faculty of mind leading to the formation of presence is often neglected when observed from the perspective of virtual reality.

Sanchez-Vives and Slater (2005), suggest that presence research should be opened up, beyond the domain of computer science and other technologically oriented disciplines. Revonsuo (1995) proposed that we should consider both – the dreaming brain and the concept of Virtual Reality, as a metaphor for the phenomenal level of organization; they are excellent model systems for consciousness research. He argues that the subjective form of dreams reveals the subjective, macro-level form of consciousness in general and that both dreams and the everyday phenomenal world may be thought of as constructed “virtual realities.”

According to Revonsuo (2006), any useful scientific approach to the problem of consciousness must consider both the subjective psychological reality and the objective neurobiological reality. In Virtual Reality it’s not just the perceptual input and the technical faculties that contribute to a stronger illusion of reality but also various psychological aspects (Lombard and Ditton, 1997; Slater, 2003, 2009) relating to one’s emotion, attention, memory, and qualia (Tye, 2009) that help mold this illusion in the mind. In the case of non-mediated illusion of reality like dreams or mental imagery, the perceptual illusion is generated internally (Kosslyn, 1994, 2005; LaBerge, 1998). The dream images and contents are synthesized to fit the patterns of those internally generated stimulations creating a distinctive context for the dream reality (DR; Hobson and McCarley, 1977; Hobson, 1988). Whether mediated or non-mediated, the illusion of reality is greatly affected by the context. “A context is a system that shapes conscious experience without itself being conscious at that time” (Baars, 1988, p. 138). Baars describes how some types of contexts shape conscious experience, while others evoke conscious thoughts and images or help select conscious percepts. In fact it’s a fine blend of perceptual and psychological illusions (explained in section The Illusion of Reality) that leads to a strong illusion of reality in one’s mind. We attempt to explore this subjective reality that is the fundamental source of experience for presence.

## Presence and Reality

With the growing interest in the field of Virtual Reality, the subject of presence has evolved to be a prime area of research. The concept of presence, as Steuer (1992) describes, is the key to defining Virtual Reality in terms of human experience rather than technological hardware. Presence refers not to one’s surroundings as they exist in the physical world, but to the perception of those surroundings as mediated by both automatic and controlled mental processes.

### Presence

Presence is a concept describing the effect that people experience when they interact with a computer-mediated or computer-generated environment (Sheridan, 1992). Witmer and Singer (1994) defined presence as the subjective experience of being in one environment (there) when physically in another environment (here). Lombard and Ditton (1997) described presence as an “illusion of non-mediation” that occurs when a person fails to perceive or acknowledge the existence of a medium in his/her communication environment and responds as he/she would if the medium were not there. Although their definition confines to presence due to a medium, they explained how the concept of presence is derived from multiple fields – communication, computer science, psychology, science, engineering, philosophy, and the arts. Presence induced by computer applications or interactive simulations was believed to be what gave people the sensation of, as Sheridan called it, “being there.” But the studies on presence progressed with a slow realization of the fact that it’s more than just “being there.” We believe that presence, whether strong or mild is the result of an “experience of reality.”

In fact “presence” has come to have multiple meanings, and it is difficult to have any useful scientific discussion about it given this confusion (Slater, 2009). There can be no advancement simply because when people talk about presence they are often not talking about the same underlying concept at all. No one is “right” or “wrong” in this debate; they are simply not talking about the same things (Slater, 2003). On the general problems in conveying knowledge due to the intersection of the conceptual, material, and linguistic representations of the same thing, there exists an attempt to explain the workings of communication and its mishaps (Schmidt, 1997a,b, 2009), which clearly states that scientists must always indicate which representation they speak of. In this article, we are mainly speaking about the phenomenon, which is the *experience* of presence.

### Reality

The term “reality” itself is very subjective and controversial. While objectivists may argue that reality is the state of things as they truly exist and is mind-independent, subjectivists would reason that reality is what we perceive to be real, and there is no underlying true reality that exists independently of perception. Naturalists argue that reality is exhausted by nature, containing nothing supernatural, and that the scientific method should be used to investigate all areas of reality, including the human spirit (Papineau, 2009). Similarly a physicalist idea is that the reality and nature of the actual world conforms to the condition of being physical (Stoljar, 2009). Reality is independent of anyone’s beliefs, linguistic practices, or conceptual schemes from a realist perspective (Miller, 2010). The Platonist view is that reality is abstract and non-spatiotemporal with objects entirely non-physical and non-mental (Balaguer, 2009). While some agree that the physical world is our reality, the Simulation Argument suggests that this perceivable world itself may be an illusion of a simulated reality (SR; Bostrom, 2003). Still others would endeavor to say that the notion of physical world is relative as our world is in constant evolution due to technological advancement; also because of numerous points of view on its acceptation (Schmidt, 2008). Resolving this confusion about theories on reality is not our primary aim and is however beyond the scope of this study. So we reserve the term “Primary Reality” to signify the reality of our real world experiences, which would be explained later in this paper.

### The illusion of reality

The factors determining the experience of presence in a virtual environment have been explored by many in different ways. For example, presence due to media has previously been reviewed as a combination of:

- Perceptual immersion and psychological immersion (Biocca and Delaney, 1995; Lombard and Ditton, 1997).
- Perceptual realism and social realism (Lombard and Ditton, 1997).
- Technology and human experience (Steuer, 1992, 1995).
- Proto-presence, core-presence, and extended-presence (Waterworth and Waterworth, 2006).
- Place illusion and plausibility illusion (Slater, 2009).

To summarize, the two main factors that contribute to the illusion of reality due to media are (1) Perceptual Illusion: the continuous stream of sensory input from a media, and (2) Psychological Illusion: the continuous cognitive processes with respect to the perceptual input, responding almost exactly how the mind would have reacted in Primary Reality. Virtual reality systems create highest levels of illusion simply because it can affect more senses and help us experience the world as if we were inside it with continuous updated sensory input and the freedom to interact with virtual people or objects. However other forms of media, like a movie (where the sensory input is merely audio-visual and there is no means to interact with the reality presented) can still create a powerful illusion if it manages to create a stronger Psychological Illusion through its content (for example a story related to one’s culture or past experiences, would excite the memory and emotional aspects). One of the obvious examples illustrating the strength of Perceptual illusion is a media that enforces stereoscopic view enhancing our depth perception (the illusion works due to the way our visual perception would work otherwise, without a medium). The resultant of the two, Perceptual Illusion and Psychological Illusion evokes an illusion of reality in the mind, although subjectively varying for each person – in strength and experience.

### The concept of “evoked reality”

We know that it’s not directly presence that we create but rather an illusion in our minds as a result of which we experience presence. When we use virtual reality systems and create convincing illusions of reality in the minds of users, they feel present in it. This illusion of reality that we evoke through different means in order to enable the experience of presence is what we intend to call “*Evoked Reality* (ER).” To explore this experience of presence we must first better understand what ER is.

**Figure 1:**
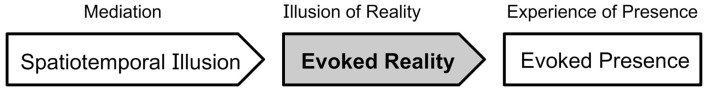
**Spatiotemporal illusion due to mediation: reality so evoked generates the experience of presence**.

As deduced earlier, all the factors influencing presence would essentially be categorized as Perceptual Illusion and Psychological Illusion. We believe that every media in a way has these two basic elements. Thus ER is a combined illusion of Perceptual Illusion and Psychological Illusion. This combined spatiotemporal illusion is what evokes a different reality in our minds (Figure 1) inducing presence.

## Evoked Reality

Even though the terms like telepresence and virtual reality are very recent, their evidence can be traced back to ancient times. The urge to evoke reality different from our Primary Reality (real world reality) is not at all new and can be observed through the evolution of artistic and scientific media throughout history. “When anything new comes along, everyone, like a child discovering the world, thinks that they’ve invented it, but you scratch a little and you find a caveman scratching on a wall is creating virtual reality in a sense. What is new here is that more sophisticated instruments give you the power to do it more easily. Virtual Reality is dreams.” Morton Heilig. (as quoted in Hamit, 1993, p. 57).

### From caves to CAVEs

**Figure 2:**
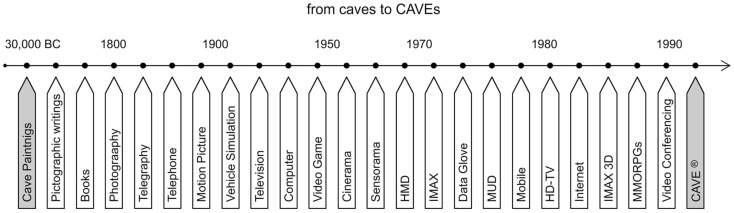
**Evolution of media: from caves to CAVEs**.

Since the beginning of civilizations, man has always tried to “express his feelings,” “convey an idea,” “tell a story” or just “communicate” through a number of different media. For example, the cave paintings and symbols that date back to prehistoric times may be considered as one of the earliest forms of media used to convey ideas. As technology progressed media evolved as well (Figure 2) and presently we are on the verge of extreme possibilities in mediation, thus equivalent mediated presence.

We all like to experience presence different from our everyday happenings. To do so, we basically find methods to create an illusion of reality different from the reality that we are familiar with. With the help of different media we have already succeeded to evoke a certain amount of presence and we further aim for an optimum level – almost similar to our real world. Every form of mediation evokes a different kind of illusory reality and hence different degrees of presence. In the early examples of research in presence, studies were conducted based on television experiences before Virtual Reality became a more prominent field of research (Hatada and Sakata, 1980). While some types of media evoke mild illusion of presence, highly advanced media like Virtual Reality may evoke stronger presence. “But we must note that the basic appeal of media still lies in the content, the storyline, the ideas, and emotions that are being communicated. We can be bored in VR and moved to tears by a book” (Ijsselsteijn, 2003). This is precisely why the reality evoked (by media) in one’s mind depends greatly on the eventual psychological illusion, although it may have been triggered initially by a perceptual illusion. Media that could evoke mild or strong presence may range from simple paintings to photos to televisions to films to interactive games to 3D IMAX films to simulation rides to immersive Virtual Reality systems.

### Evoked reality

Evoked Reality is an illusion of reality, different from our Primary Reality (Physical Reality as referred in previous studies). ER is a transient subjective reality created in our mind. In the case of ER due to media, the illusion persists until an uninterrupted input of perceptual stimuli (causing perceptual illusion) and simultaneous interactions (affecting the psychological illusion) continue to remain. The moment at which this illusion of ER breaks due to an anomaly is when we experience what is called a “Break in Presence (BIP)” (Slater and Steed, 2000; Brogni et al., 2003). Thus a BIP is simply an immediate result of the “Break in Reality (BIR)” experienced. Different kinds of media can evoke realities of different qualities and different strengths in our minds for different amount of time. It’s an illusion of space or events, where or during which we experience a sense of presence. Thus, it is this ER in which one may experience Evoked Presence (EP).

### Evoked presence

Depending on the characteristics of ER, an experience of presence is evoked. To be more specific this illusion of presence created by ER, we would like to refer to as EP. In this paper, the term “EP” would imply the illusion of presence experience (the sense of presence), while the term “presence” would be reserved for experience of presence in its broad sense (real presence and the sense of presence). *EP is the spatiotemporal experience of an ER*. We could say that so far it’s through the media like highly immersive virtual reality systems, that we were able to create ER that could evoke significantly strong EP.

### Media-evoked reality and self-evoked reality

As we saw before, ER is a momentary and subjective reality created in our mind due to the Perceptual Illusion and Psychological Illusion imposed by a media. It is clear that due to ER induced through media like Virtual Reality we experience an EP. This illusion of reality evoked through media, we would like to call “*Media-Evoked Reality*” or Media-ER.

As mentioned earlier, it’s not just through the media that one can evoke an illusion of reality. The illusion can as well be endogenously created by our mind evoking a seemingly perceivable reality; whether merely observable or amazingly deformable; extremely detailed or highly abstract; simple and familiar or bizarrely uncanny. Thus to fully comprehend the nature of presence, we must study this category of ER that does not rely on media. In fact, we always or most often undergo different types of presence without mediation. Sanchez-Vives and Slater (2005) proposed that the concept of presence is sufficiently similar to consciousness and that it may help to transform research within domains outside Virtual Reality. They argue that presence is a phenomenon worthy of study by neuroscientists and may help toward the study of consciousness. As rightly put by Biocca (2003), where do dream states fit in the two pole model of presence (Reality-Virtuality Continuum)? The psychological mechanisms that generate presence in a dream state have to be at least slightly different than psychological mechanisms that generate presence in an immersive, 3D multimodal virtual environment. Dreaming, according to Revonsuo (1995) is an organized simulation of the perceptual world and is comparable to virtual reality. During dreaming, we experience a complex model of the world in which certain types of elements, when compared to waking life, are underrepresented whereas others are over represented (Revonsuo, 2000). According to LaBerge (1998), theories of consciousness that do not account for dreaming must be regarded as incomplete. LaBerge adds, “For example, the behaviorist assumption that ‘the brain is stimulated always and only from the outside by a sense organ process’ cannot explain dreams; likewise, for the assumption that consciousness is the direct or exclusive product of sensory input.” It is very clear that one can think, imagine, or dream to create a reality in his mind without the influence of any media whatsoever. This reality evoked endogenously, without the help of an external medium, we would like to call “*Self-Evoked Reality*” or Self-ER (implying that the reality evoked is initiated internally by the mind itself).

Ground-breaking works by Shepard and Metzler (1971) and Kosslyn (1980, 1983) in the area of Mental Imagery provide empirical evidence of our ability to evoke images or imagine stimuli without actually perceiving them. We know that Perceptual and Psychological Illusion are factors that affect Media-ER and corresponding EP. We believe that Self-ER essentially has Psychological Illusion for which the Perceptual element is generated internally by our mind. By generally overlooking or occasionally completely overriding the external perceptual aspects (sensorimotor cues), our mind endogenously creates the Perceptual Illusion required for the ER. It’s evident in the case of dreaming which according to LaBerge (1998), can be viewed as the special case of perception without the constraints of external sensory input. Rechtschaffen and Buchignani (1992) suggest that the visual appearance of dreams is practically identical with that of the waking world. Moreover, Kosslyn’s (1994, 2005) work show that there are considerable similarities between the neural mappings for imagined stimuli and perceived stimuli.

Similar to Media-ER, one may feel higher or lower levels of presence in Self-ER, depending on the reality evoked. A person dreaming at night may feel a stronger presence than a person who is daydreaming (perhaps about his first date) through an on-going lecture with higher possibilities of BIRs. According to Ramachandran and Hirstein (1997) we occasionally have a virtual reality simulation like scenario in the mind (although less vivid and generated from memory representations) in order to make appropriate decisions in the absence of the objects which normally provoke those qualities. However, the vividness, strength, and quality of this internally generated illusion may vary significantly from one person to another. For example, the intuitive “self-projection” phenomenon (Buckner and Carroll, 2007; personal internal mode of mental simulation, as they refer to it) that one undergoes for prospection will certainly differ in experience and qualia from another person. It is a form of Self-ER that may not be as strong or prolonged as a picturesque dream, but strong enough to visualize possible consequences. It is clear that ER is either the result of media or induced internally. This dual (self and media evoking) nature of ER directs us toward a fresh perceptive – three poles of reality.

## Three Poles of Reality

As we move further into the concept of ER and EP, we would like to define the three poles of reality to be clearer and more objective in the explanations that follow. Reality, as discussed earlier (in subsection Simulated Reality), has always been a term interpreted with multiple meanings and theories. To avoid confusion we would like to use an impartial term – “Primary Reality,” which would refer to the “experience” of the real world (or what we call physical world). It is the spatiotemporal reality in our mind when we are completely present in the real world. It would mean that any reality other than Primary Reality is a conscious experience of illusion of reality (mediated or non-mediated), or more precisely – ER.

### Presence and poles of reality

**Figure 3:**
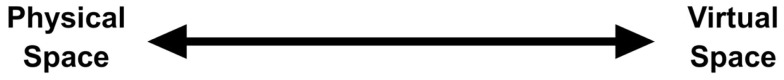
**The standard two pole model of presence**.

Inherited from early telerobotics and telepresence research, the two pole model of presence (Figure 3) suggests that presence shifts back and forth from physical space to virtual space. Research on presence has been dominated ever since by this standard two pole psychological model of presence which therefore requires no further explanation.

Biocca (2003) took the study of presence model one step further. According to the model he proposed, one’s spatial presence shifts between three poles of presence: mental imagery space, the virtual space, and the physical space. In this three pole graphic model, a quasi-triangular space defined by three poles represented the range of possible spatial mental models that are the specific locus of an individual user’s spatial presence. His Model of presence attempted to offer a parsimonious explanation for both the changing loci of presence and the mechanisms driving presence shifts. Though the model explained the possibilities of presence shifts and varying levels of presence, it is vague about certain aspects of reality. It did not clarify what happens when we experience an extremely low level of presence (at the center of the model). How or why do we instantly return to our Primary Reality (in this model – Physical Space) as soon as a mediated reality or a DR is disrupted (Even though we may have entirely believed to be present in the reality evoked during a vivid dream)? Moreover it took into account only the spatial aspects but not the temporal aspects of shifts in presence.

**Figure 4:**
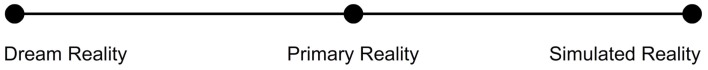
**Three pole reality model**.

We would like to define three poles of reality from the perspective of ER. The Three Pole Reality Model (Figure 4) may help overcome the theoretical problems associated with presence in the standard two pole model of presence as well as the model proposed by Biocca. According to us it’s the shifts in the type of reality evoked that create respective shifts in the level of presence evoked. For example if one experiences a highly convincing ER during a virtual reality simulation, he/she would experience an equivalently strong EP until a BIR occurs. The three poles of reality that we define are:

- DR (Threshold of Self-ER)
- Primary Reality (No ER)
- SR (Threshold of Media-ER)

#### Primary reality

**Figure 5:**
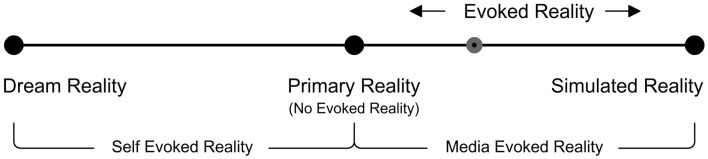
**Three poles of reality: evoked reality constantly shifts between them**.

Primary reality refers to the reality of our real world. In Primary reality, the experience evoking stimulation arrives at our sensory organs directly from objects from the real world. We maintain this as an ideal case in which the stimulus corresponds to the actual object and does not deceive or misinform us. For instance, imagine yourself running from a tiger that is chasing you. It’s very near and is about to pounce on you. You scream in fear, and wake up to realize that you are safe in your bed, like every morning. You know for sure that this is the real world and the chasing tiger was just a part of the DR that your mind was in, some time before. So, *Primary Reality is our base reality to which we return when we are not in any ER*. In other words, when a BIR occurs, we come back to Primary Reality. Thus, as we can see in Figure 5, any point of reality other than Primary Reality is an ER. We could say that it’s this Primary Reality that we rely on for our everyday activities. It’s the reality in which we believe that we live in. Our experiences in this Primary Reality may form the basis for our experiences and expectations in an ER. For example, our understanding of the real world could shape how we experience presence in an immersive virtual reality environment, or even in a Dream. We could suppose that it’s the Primary Reality in which one believes this paper exists, or is being read.

#### Simulated reality

In the case of Media-ER, an experience similar to Primary Reality is attempted to be achieved by interfering with the stimulus field, leading to an illusion of reality. For example virtual reality uses displays that would entirely mediate our visual perception in a manner that our head or eye movements are tracked and updated with appropriate images to maintain this illusion of receiving particular visual stimuli from particular objects. SR would be the most compelling and plausible reality that could ever be achieved through such mediations. It would be the reality evoked in our mind under the influence of a perfectly simulated virtual reality system. It’s the ultimate level that virtual reality aims to reach someday. At the moment an immersive virtual reality system, like flight simulators would be able to create ER considerably close to this pole. Its effectiveness is evident in the fact that pilots are able to perfectly train themselves being in that ER created by the simulator, helping them eventually to directly pilot a real plane. However, in the hypothetical condition of a perfectly SR our mind would completely believe the reality evoked by the simulation medium, and have no knowledge of the parent Primary Reality (Putnam, 1982; Bostrom, 2003). In this state, it would be necessary to force a BIR to bring our mind back to Primary Reality. *A Perfect SR is the Media-ER with strongest presence evoked and will have no BIRs*.

#### Dream reality

In the case of Self-ER, the external perceptual stimuli are imitated by generating them internally. DR is an ideal mental state in which we almost entirely believe in the reality experienced, and accept what is happening as real. It does not return to the Primary Reality unless a BIR occurs. For instance, in the case of our regular dreams, the most common BIR would be “waking up.” Although internally generated, dream states may not be completely divorced from sensorimotor cues. There can be leakage from physical space into the dream state (Biocca, 2003). The experienced EP during a strong Dream can be so powerful that even the possible anomalies (causing BIRs) like external noises (an alarm or phone ringing) or even elements from physical disturbances (blowing wind, temperature fluctuations) may be merged into the DR, so as to sustain this ER for as long as possible. *A Perfect DR is a Self-ER with the strongest presence evoked and will have no BIRs* (similar to SR on the media side).

### Presence shifts and presence threshold

We are often under the effect of either Media or Self-ER. Imagine that we are not influenced by any mediation, nor any kind of thoughts, mental imagery, or dreams and our mind is absolutely and only conscious about the Primary Reality. In such an exceptional situation we would supposedly feel complete presence in the Primary Reality. Thus we presume that this perfect Primary Reality-Presence (or “real presence” as some may call) is the threshold of presence one’s mind may be able to experience at a point of time. It is clear that we can experience presence either in Primary Reality or in an ER. We cannot consciously experience presence in two or more realities at the same time, but our mind can shift from one reality to another voluntarily or involuntarily, thus constantly shifting the nature and strength of the presence felt. As pointed out by Garau et al. (2008), presence is not a stable experience and varies temporally. They explain how even BIPs could be of varying intensities. They also try to illustrate using different presence graphs the phenomenon of shifting levels of presence with the course of time and how subjective the experience is for different participants. Media like virtual reality aims to achieve the Presence Threshold at which one’s mind might completely believe the reality evoked. Though we have not however achieved it, or may never do, theoretically it’s possible to reach such a level of SR. Similarly if one experiences a Perfect Dream without any BIR, he/she would be at this threshold of presence exactly like being in the Primary Reality. *SR and DR are the two extreme poles of reality at which the EP is at its threshold*. These presence shifts due to the shifting of reality between these poles is something that we seldom apprehend, although we always experience and constantly adapt to them. In the following section we attempt to represent this phenomenon with a schematic model that would help us examine presence and reality from a clearer perspective.

## Reality-Presence Map

**Figure 6:**
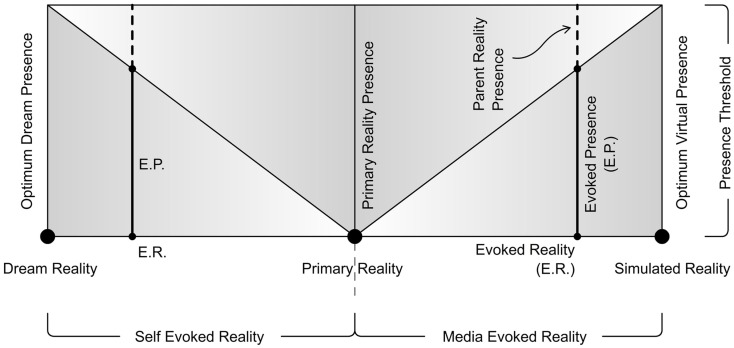
**Reality-presence map**.

Based on the three poles of reality and Presence Threshold we would like to propose the Reality-Presence Map (Figure 6). This map is a diagram of the logical relations between the terms herein defined. At any point of time one’s mind would be under the influence of either a Media-ER or a Self-ER when not in the Primary Reality (with no ER at all). Between the poles of reality, ER would constantly shift evoking a corresponding presence EP. As we can see in the map there is always a sub-conscious Parent Reality-Presence corresponding to the EP. This Parent Reality-Presence is very important as it helps our mind to return to the Primary Reality once the illusion of ER discontinues (or a BIR occurs). For a weaker EP, the Parent Reality-Presence is stronger (although experienced sub-consciously). When the ER manages to evoke very strong presence, the strength of Parent Reality-Presence drops very low (almost unconscious) and we start to become unaware of the existence of a Primary Reality; which is what an excellent immersive virtual reality system does. The shifting of presence is closely related to our attention. As soon as our attention from the ER is disrupted (predominantly due to interfering external perceptual elements), our attention shifts to the parent reality-presence sliding us back to Primary Reality (thus breaking our EP).

At the extreme poles, we would experience an Optimum Virtual Presence in a SR and similarly an Optimum Dream Presence in a DR. At these extreme points one may completely believe in the illusion of reality experienced almost or exactly like it is our Primary Reality, without the knowledge of an existing Parent Reality. At such a point, possibly a very strong BIR should be forced to bring one back to the parent Primary Reality. Experiencing a strong DR is one such example which many would relate to. During a very compelling but frightening dream, “waking up” acts as a very strong BIR, helping in the desperate attempt to leave the DR. After such a sudden and shocking change in reality most often our mind takes time to adjust back to the Primary Reality where everything would slowly turn normal and comforting.

Whenever there is an ER, the EP part of the presence (in the map) is what has our primary attention, and thus is the conscious part. Hence, the higher the EP, the lesser we are aware of our parent reality. Evidence of the sub-conscious Parent Reality-Presence can be observed in our experience of any media that exists today. Many studies have shown that in virtual environments, although the users behaved as if experiencing the real world, at a sub-conscious level they were certain that it was indeed “not” real. BIPs (that are used to measure presence) are in fact triggered by shifts in attention from the virtual world to the real world. For instance, virtual reality systems that help visually surround us completely with a virtual environment, elevates our presence (compared to a panorama view or television with visible frame boundaries) as our chances of shifting attention toward the real world drastically reduce in such higher levels of immersion (Grau, 2004; Slater, 2009). Since ER is a subjective feeling, it can never be measured or even compared truthfully. This is the reason why we depend on the measurement of presence EP to determine if a system creates a stronger or weaker ER. Since the strength of presence itself is relative, the best way to measure is to compare between systems in similar context. “The illusion of presence does not refer to the same qualia across different levels of immersion. The range of actions and responses that are possible are clearly bound to the sensorimotor contingencies set that defines a given level of immersion. It may, however, make sense to compare experience between systems that are in the same immersion equivalent class” (Slater, 2009).

A major task for empirical consciousness research is to find out the mechanisms which bind the experienced world into a coherent whole (Revonsuo, 1995). This map provides a framework where the various experiences of ER could be mapped. Note that this map is not a “graph” that shows the strength of EP as directly proportional to the strength of ER. In fact it would help us represent every possible kind of ER as a point fluctuating between the two extreme poles of reality, with its respective strength of EP. We may refer to ER as stronger or weaker, when its qualia evoke stronger or weaker EP respectively. The Reality-Presence Map shows that if we can skillfully manipulate these qualia of ER (although subjective to each individual) bringing it closer to either of the two extreme poles, we may be able to evoke higher levels of EP. We should also note that, in order to introduce its basic concept, the Reality-Presence Map is presented here in a flattened two-dimensional manner. In the later sections we will illustrate how this map attempts to account for different experiences which were unable to be explained by previous presence models.

### Subjectivity of evoked reality

As a matter of fact, the same mediation can create different subjective ER for different users depending on their personal traits. For example, two users reading the same book, or playing the same video game, or using the same Virtual Reality system would experience presence in an entirely different manner. EP (especially evoked by a medium) may be affected by one’s knowledge related to the context, degree of interest, attention, concentration, involvement, engagement, willingness, acceptance, and emotional attributes making it a very subjective experience. This is precisely why it is difficult to evaluate the efficiency of a particular Virtual Reality system by means of presence questionnaires. In fact many researchers confuse few of these terms above, with the concept of presence.

**Figure 7:**
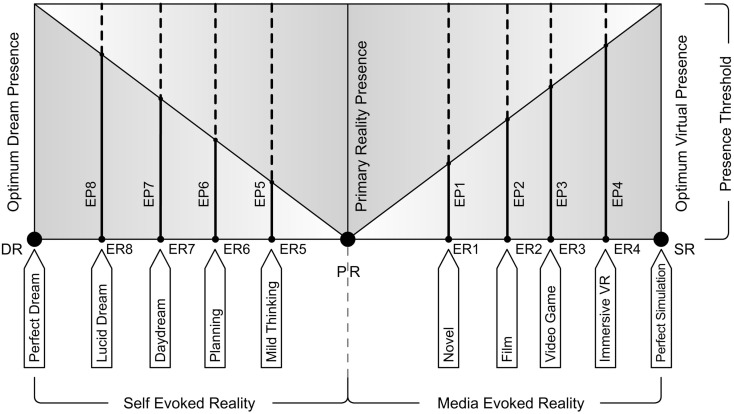
**An example range of Media-ER and Self-ER experiences mapped on reality-presence map, for an individual, that would occur at various points in time**.

Therefore, to locate ER on the map, we have to examine “presence.” In fact finding reliable ways to measure presence has been a pursuit among many virtual reality and communication media researchers. In order to lead to testable predictions, we would rely on currently evolving measuring and rating systems, so as to determine an objective scale for presence (from Primary Reality to each extreme pole). Presently existing measuring techniques include questionnaires like “presence questionnaire” (Witmer and Singer, 1998; Usoh et al., 2000), ITC-SOPI questionnaire (Lessiter et al., 2001), SUS questionnaire (Slater et al., 1994, 1995), analysis of BIPs (Slater and Steed, 2000; Brogni et al., 2003), objective corroborative measures of presence like psycho-physiological measures, neural correlates, behavioral measures, task performance measures (Van Baren and Ijsselsteijn, 2004), to mention a few. We can certainly predict the positions of different everyday experiences for a person in general (Figure 7); however it could be tested in the future only using above mentioned methods of measuring presence.

In virtual reality, distinction between “presence” and “immersion” has been made very clear previously in (Slater, 1999, 2003). Though immersion (which is discussed extensively in the domain of virtual reality) is one of the significant aspects of EP, it falls under the technical faculty of a mediated system. “Immersion (in perceptual sense) provides the boundaries within which Place Illusion can occur” (Slater, 2009). Detailed aspects of presence related to immersive virtual reality are also discussed in (Slater et al., 2009). The characteristics like involvement, engagement, degree of interest, emotional response, may seem similar to presence, but are in fact different elements that may influence or be influenced by EP. The psychological impact of content, i.e., good and bad, exciting and boring, depends to a large extent on the form in which it is represented (Ijsselsteijn, 2003). Thus one of the most important aspects of Media-ER is its context. In most cases it forms a reference in one’s mind to how they may experience ER and hence the presence evoked. For example, in some contexts, especially in art and entertainment, it would invoke a “genre” that plays a major role in its communication. The context (whether artistic expression, communication, entertainment, medical application, education, or research) should be a core concern while designing a Virtual Reality System, in order to bring about a subjectively higher quality of ER. A descriptive account on the importance of context in Self-ER is given by Baars (1988). With examples of different sources and types (perceptual and conceptual) of contexts, he demonstrates how unconscious contexts shape conscious experience. In addition, he explains the importance of attention, which acts as the control of access to consciousness. Attention (in both Media-ER and Self-ER) can direct the mind toward or away from a potential source of qualia. The experience of an ER therefore depends also on the voluntary and involuntary characteristics of one’s attention.

According to the concept, our presence shifts continuously from one ER to another and does not require passing through Primary Reality to move from one side to another. This map does not provide a temporal scale *per se*. However in future (with the advancements in presence measurement techniques), the map can be used to trace presence at different times to study the temporal aspects of presence shifts.

### Evoked reality within evoked reality

There is an important question that arises now. How can we account for our thoughts or mental imagery experiences during VR simulations, games, movies, or most importantly books? It is the phenomena of experiencing Self-ER during a Media-ER experience.

#### Self-ER within media-ER

**Figure 8:**
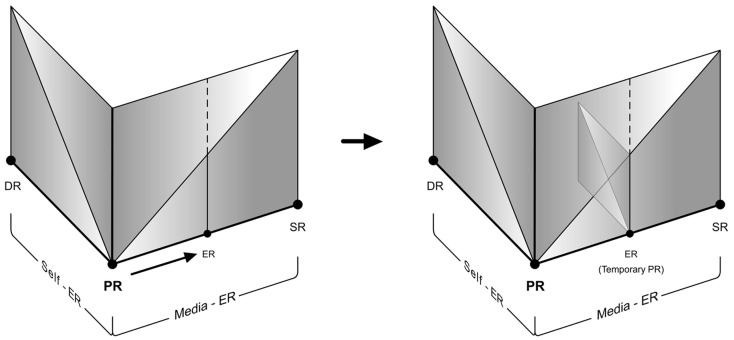
**An example of how Media-ER would temporarily act as a version of primary reality**.

**Figure 9:**
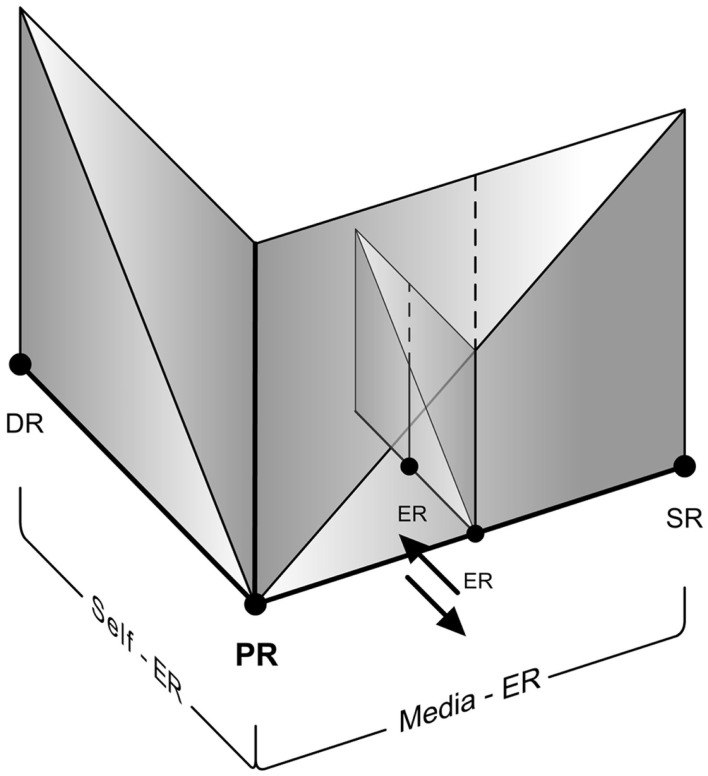
**An example of presence shift due to Self-ER within Media-ER (for e.g., thinking within a virtual environment)**.

Whenever we experience an ER, our mind is capable of temporarily presuming it as the parent reality and reacting accordingly. The better the ER and stronger the EP, the easier it is for our mind to maintain the illusion. In such states Media-ER is experienced as a temporarily form of Primary Reality, and we are able to experience Self-ER within it. In fact that is the core reason why virtual reality systems and virtual environments work. This phenomenon is clearly displayed in such experiences, where the users require thinking, planning, and imagination in order to navigate in the virtual world, just like they would do in the real world. Below, it is demonstrated how this phenomenon may be represented with respect to the Reality-Presence Map (Figures 8 and 9). This scenario will ultimately be classified under Media-ER.

#### Self-ER triggered during media-ER

**Figure 10:**
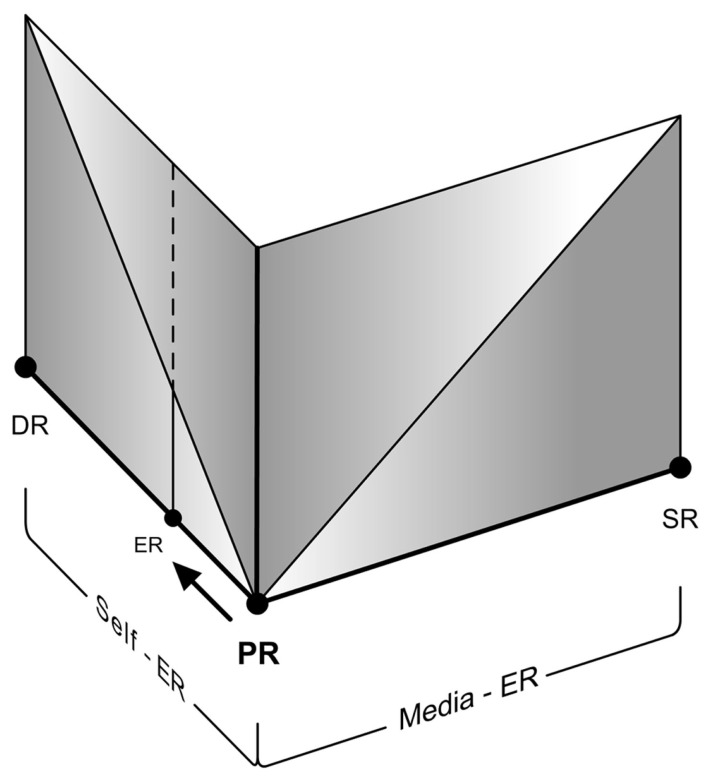
**The usual case of presence shift from primary reality to Self-ER**.

“Self-ER within Media-ER” should be distinguished from the phenomenon of “Self-ER triggered during Media-ER.” This is similar to a well-known case of Self-ER – the phenomenon of mind-wandering that temporarily detaches us from the Primary Reality. It is otherwise known as “task unrelated thought,” especially with respect to laboratory conditions. Smallwood et al. (2003) define it as the experience of thoughts directed away from the current situation. It is in fact a part of (and closely related to) our daily life experiences (Smallwood et al., 2004; McVay et al., 2009). Although studies on mind-wandering are principally focused on shifts between Self-ER and tasks relating to Primary Reality (falling under usual case of Self-ER experience – Figure 10), we propose that they are applicable to similar cases in Media-ER as well. It has been suggested that this involuntary experience may be both stable and a transient state. That means we can experience a stable EP during mind-wandering or an EP oscillating between the Self-ER, Media-ER, and the Primary Reality.

**Figure 11:**
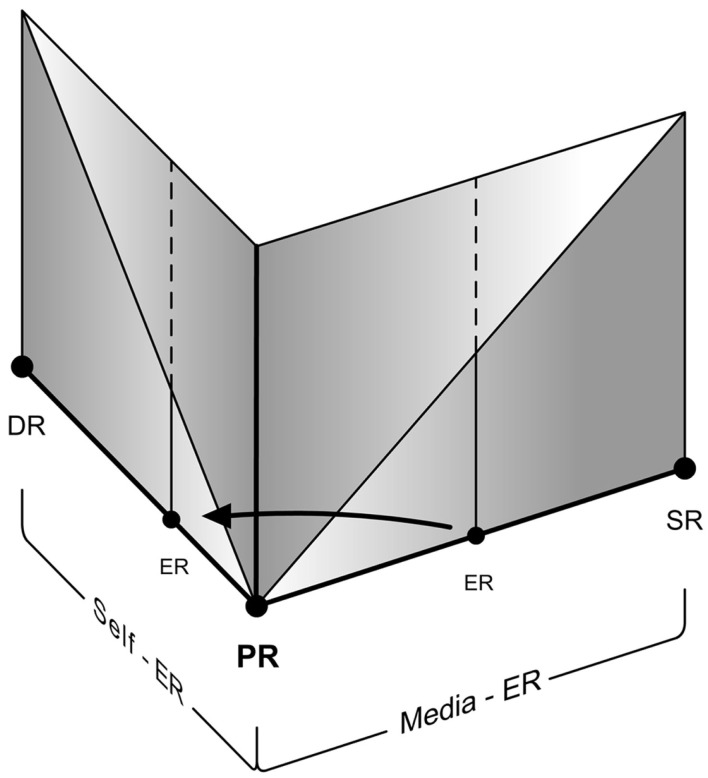
**An example of presence shift toward Self-ER triggered during Media-ER**.

Therefore, when an unrelated Self-ER is triggered while experiencing a Media-ER (or when Self-ER within Media-ER traverse the presence threshold and becomes unaware of the Media-ER itself), it should be considered under the case of Self-ER (Figure 11).

## Discussion

Our attempt was a novel idea, to fit together different concepts regarding presence into a single coherent graphical representation. Although this concept of ER and EP along with the proposed map provides us a simplified way to look at reality and presence, it raises plenty of questions. Can the experience of an altered state of consciousness (ASC) like hallucination, delusion, or psychosis due to mental disorders be a kind of Self-ER? Revonsuo et al. (2009) redefines ASC, as the state in which consciousness relates itself differently to the world, in a way that involves widespread misrepresentations of the world and/or the self. They suggest that, to be in an ASC is to deviate from the natural (world-consciousness) relation in such a way that the world and/or self tend to be misrepresented (as evident in reversible states like dreaming, psychotic episodes, psychedelic drug experiences, epileptic seizures, and hypnosis). According to Ramachandran and Hirstein (1997) we have internal mental simulations in the mind using less vivid perceptual attributes, in the absence of the regular external sensory inputs. If they possessed full-strength perceptual quality, that would become dangerous leading to hallucinations. They argue that in cases like temporal lobe seizures, this illusion (Self-ER) may become indistinguishable to real sensory input losing its revocability and generating incorrect sense of reality (creating a permanent ER situation that makes it difficult to return to Primary Reality). So can hallucinations due to Self-ER be compared to Augmented Reality due to Media-ER?

In contrast to Presence, is there an “Absence” and do we experience that? If so, how? Can it be compared to a dreamless sleep? Can Presence Threshold itself be subjective and differ from person to person? With reference to the Reality-Presence Map, is there a possibility of an experience analogous to uncanny valley when ER is nearest to the two extreme poles? Is this the reason why many experience anomalies during exceptionally vivid nightmares or lucid dreams? Similarly on the Media-ER side, can simulator sickness due to inconsistencies during virtual reality simulations be compared to this phenomenon? Other than the obvious difference between Media-ER and Self-ER that was discussed before, they have another main differentiation. In most cases of Media-ER, multiple users could share the experience of a common ER at the same time (naturally, with subjective differences, especially due to psychological illusion). While in the case of Self-ER, every person’s mind experiences unique ER. Thus a Dream is typically an individual experience (as far as our present technological advancements and constraints suggest), while SR may be shared.

**Figure 12:**
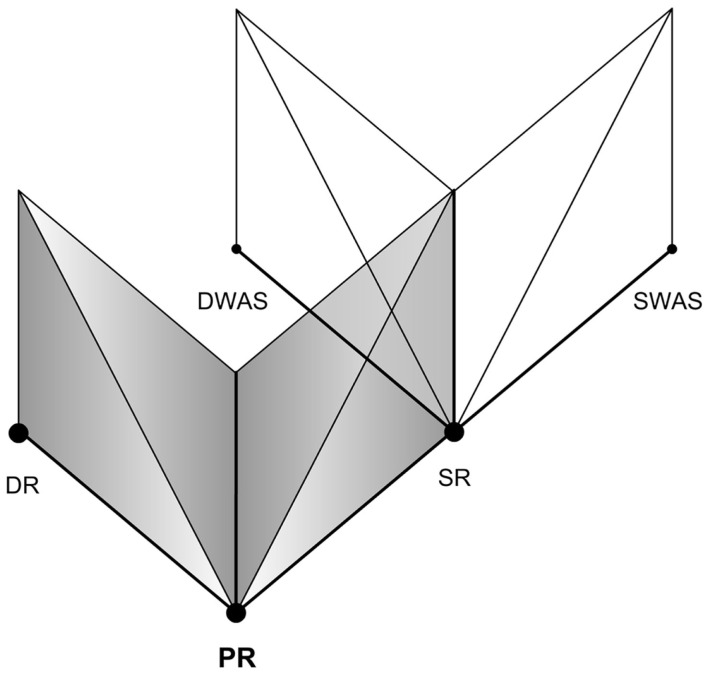
**Simulation within a simulation**.

**Figure 13:**
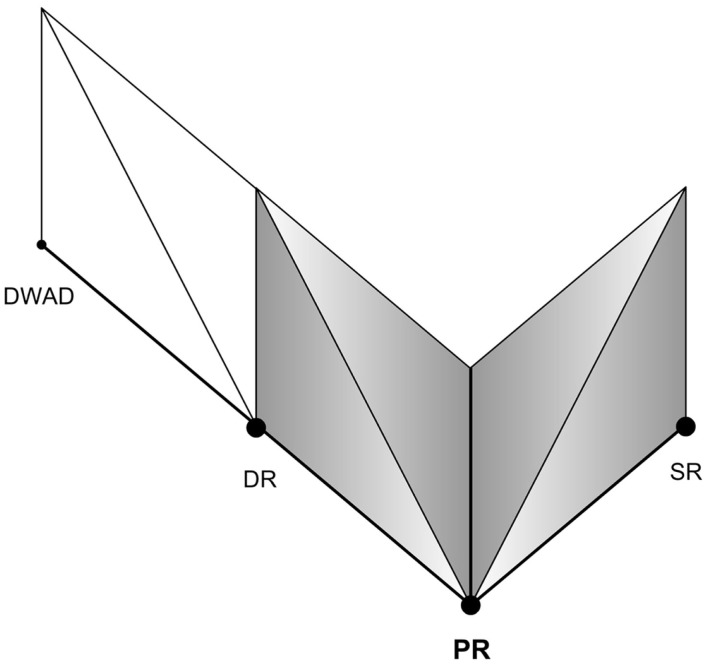
**Dream within a dream**.

Furthermore, the Reality-Presence Map helps us investigate into potential ideas on Reality, for instance the possibility of Simulation within a Simulation (SWAS). The Map could be extended to and be applicable for any level of reality, in which we believe there’s a Primary Reality – the base reality, to which we return to in case of absence of any form of ER. Let’s imagine that someday we achieve a perfect SR. As per our proposition, one’s mind would accept it as the Primary Reality as long as the experience of presence continues (or till a “BIR” occurs). It would imply that at such a point, one can experience presence exactly as in the Primary Reality. In this perfect SR if one experiences Media-ER (e.g., virtual reality) or Self-ER (e.g., dream), as soon a BIR occurs they return back to it since it’s the immediate Parent Reality. Figure 12 attempts to illustrate such a situation with DR and SR as two orthogonal Poles of Reality. Similarly in the Self-ER side, one’s mind could experience a Dream within a Dream (DWAD). When one wakes up from such a dream, he could find himself in the parent DR from which he would have to wake up again into the Primary Reality. Can this be how people experience such false awakenings [a hallucinatory state distinct from waking experience (Green and McCreery, 1994)]? Figure 13 attempts to illustrate such a situation of DWAD.

**Figure 14:**
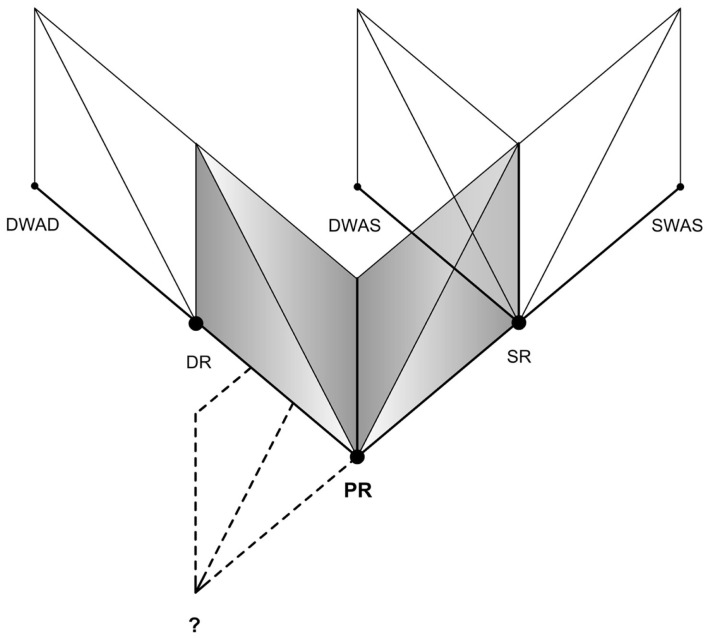
**Reality continuum (illustrating the levels of reality)**.

In fact it makes us curious about the even bigger questions. Can there be an ultimate reality beyond Primary Reality or even beyond the scope of this map. The Simulation argument claims that we are almost certainly living in a computer simulation (Bostrom, 2003), in which case what we believe to be our Primary Reality might itself be a SR [similar to Brains in a vat scenario (Putnam, 1982)]. Metzinger (2009) proposes that our experience of the Primary Reality is deceptive and that we experience only a small fraction of what actually exists out there. He suggests that no such thing as “self” exists and the subjective experience is due to the way our consciousness organizes the information about outside world, forming a knowledge of self in the first person. He claims that everything we experience is in fact a SR and the on-going process of conscious experience is not so much an image of reality as an “ego tunnel” through reality. So, is our Primary Reality in fact the base reality? Or are we always under an ER of some kind? Figure 14 attempts to put together different levels of reality as a Reality Continuum. It would make us wonder if it’s probable, to how many levels would one be able to go? Do we already visit them unknowingly through our dreams? Would the levels of reality in the figure be represented as a never ending fractal structure? In any case, will we be able to understand someday all these aspects of our experience of reality?

## Conclusion

In this paper we explored presence and different elements that contribute to it. Presence is not just “being there” but a combination of multiple feelings and most importantly “experiencing the reality.” The two main factors affecting presence due to mediation are Perceptual Illusion and Psychological Illusion. These factors evoke an illusion of reality in our mind in which we feel presence. We are constantly subjected to such illusions of reality, during which we experience presence differently from that of our apparent real world. This illusion of reality is called ER.

Evoked Reality is not just media-evoked but can also be self-evoked. Media-ER may range from the mild effect of a painting to an extremely plausible immersive Virtual Reality experience while a Self-ER may range from a simple thought to an exceptionally believable DR (the strength of ER may not necessarily be in the same order, as it depends on one’s qualia and personal characteristics). This dual nature of ER led us to define three poles of reality: primary reality – the unaltered and unmediated Real World, SR – the ultimate Media-ER (a perfect Virtual Reality condition) and DR – the ultimate Self-ER (a perfect dream condition). *Thus ER is an illusion of reality formed in our mind, which is different from Primary Reality*. It’s a combined illusion of space and events, or at least one of them. It is in this ER, one would experience presence. Thus *EP is the spatiotemporal experience of an ER*.

The proposed Reality-Presence Map attempts to graphically illustrate the concept of ER and EP. This map provides a framework where the various experiences of ER could be mapped. The subjectivity of ER qualia and how these subjective factors affect Media-ER and EP were explained. The idea of Presence Threshold was also explored which formed the basis for different levels of EP and temporal Presence Shifts. Different possibilities like SWAS and DWAD conditions were discussed with respect to the proposed model. However certain elements still demand clarifications to fill in the theory. The concept presented here is an inception of a potential future research. We believe that ER and the proposed Reality-Presence Map could have significant applications in the study of presence and most importantly in exploring the possibilities of what we call “reality.”

## Conflict of Interest Statement

The authors declare that the research was conducted in the absence of any commercial or financial relationships that could be construed as a potential conflict of interest.
